# Herbal Medicines for Irinotecan-Induced Diarrhea

**DOI:** 10.3389/fphar.2019.00182

**Published:** 2019-03-29

**Authors:** Liu Tang, Xiaolei Li, Liping Wan, Yao Xiao, Xin Zeng, Hong Ding

**Affiliations:** Key Laboratory of Combinatorial Biosynthesis and Drug Discovery, Ministry of Education, School of Pharmaceutical Sciences, Wuhan University, Wuhan, China

**Keywords:** chemotherapy-induced diarrhea, irinotecan, herbal formulations, phytochemicals, mini-review

## Abstract

Irinotecan (CPT-11), a water-soluble derivative of camptothecin, belongs to the class of DNA topoisomerase I inhibitors and has been approved worldwide for the treatment of advanced colorectal cancer, lung cancer, and malignant lymphoma. Although CPT-11-based chemotherapy is widely used, severe gastrointestinal (GI) toxicity, especially late-onset diarrhea, is a common adverse reaction, limiting clinical application of the drug. The incidence of grade 3 or 4 diarrhea is high, with 20–40% of CPT-11-treated patients experiencing this adverse effect. High-dose loperamide and octreotide are generally recommended for treatment of CPT-11-induced diarrhea. However, in clinical practice, loperamide is associated with a significant failure rate and the beneficial effects of octreotide are controversial. An accumulating number of recent studies have suggested that medicinal herbs and their derived phytocompounds may be effective complementary treatments for CPT-11-induced diarrhea. In this mini-review, we briefly summarize currently available literatures regarding the formulae and herbs/natural products used as adjuvants in animal and clinical studies for the treatment of diarrhea caused by CPT-11.

## Introduction

Diarrhea is one of the most common and dose-limiting toxicities associated with a variety of chemotherapeutic agents, including fluorouracil (FU), capecitabine, and CPT-11, which are used to treat cancer. These chemotherapeutic agents induce diarrhea in 50–80% of patients when used alone or in combination (Benson et al., 2004; Sharma et al., 2005; Stein et al., 2010). Chemotherapy-induced diarrhea (CID), even low grade (1 or 2), remarkably interferes with anticancer treatment, leading to treatment delays (28–71% of patients), dose reductions (22–45% of patients), or complete treatment discontinuation (3–15% of patients) (Arbuckle et al., 2000; Arnold et al., 2005; Dranitsaris et al., 2005; McQuade et al., 2016). Severe CID (grade 3 or 4) causes significant dehydration, electrolyte imbalance, and nutritional deficiencies, which are linked to early death in roughly 5% of patients (Rothenberg et al., 2001; Andreyev et al., 2014).

CPT-11 is an established and highly effective chemotherapeutic agent used for the treatment of colon, colorectal, lung, pancreatic, as well as other types of cancer (Rosen, 1998; Mukai et al., 2009; Hayashi et al., 2013; Satouchi et al., 2014). However, severe and unpredictable dose-limiting toxicities, such as neutropenia, myelosuppression, and GI toxicity, represent a significant barrier to clinical use (Kakolyris et al., 2001; Benson et al., 2004). Persistent and severe CPT-11-associated diarrhea is debilitating and can be life-threatening. Notably, octreotide and high-dose loperamide, recommended to relieve severe late-onset diarrhea, are non-specific and often produce unsatisfactory results (Barbounis et al., 2001; Benson et al., 2004; Hoff et al., 2014). There is a great interest in finding more effective modulating agents that either alleviate delayed-onset diarrhea induced by CPT-11 chemotherapy and/or enhance antitumor efficacy.

Herbal medicines are currently of great interest to physicians because of their potential to minimize side effects of cancer treatments and improve patients’ quality-of-life (QOL). Botanicals have been widely used in the United States, Japan, Korea, and China, either as mono-therapies or as adjuvants to existing conventional therapeutics (Yamashita et al., 2002; Hori et al., 2008; Park et al., 2012; Rashrash et al., 2017). In recent years, several herbal medicines and phytochemicals have been investigated in animal models and clinical trials to assess their effects on CPT-11-induced diarrhea. This mini-review focuses on the use of medicinal herbs and their bioactive constituents for the treatment or prevention of CPT-11-induced diarrhea and their underlying biochemical and cellular mechanisms.

## CPT-11 Associated Diarrhea

The biochemical mechanisms for CID are not fully understood, but are believed to involve inflammation, secretory dysfunction, and GI dysmotility (McQuade et al., 2016). CPT-11-induced GI toxicity differs from that of other antitumor regimens in that it causes early-onset (onset ≤ 24 h after administration) and late-onset diarrhea (onset > 24 h after administration) (Richardson and Dobish, 2007).

The underlying pathogenesis for early-onset diarrhea most likely involves inhibition of acetylcholinesterase activity. Early-onset diarrhea can be ameliorated or prevented by prophylactic or therapeutic intravenous or subcutaneous administration of 0.25 mg to 1 mg of atropine (Gandia et al., 1993; Bleiberg and Cvitkovic, 1996; Yang et al., 2005). One proposed mechanism for CPT-11-induced late-onset diarrhea is intestinal exposure to SN-38 (7-ethyl-10-hydroxycamptothecin), the major active metabolite of CPT-11, resulting in colonic and intestinal mucosal damage (Hecht, 1998). Based on *in vivo* CPT-11 metabolic pathways (Stein et al., 2010; Swami et al., 2013), SN-38-induced diarrhea is influenced by carboxylesterase (Ahmed et al., 1999), bacterial β-glucuronidase (Kehrer et al., 2001), and UDP-glucuronosyltransferase (UGT) (Smith et al., 2006), all of which lead to accumulation of the toxic metabolite SN-38 in the intestines. CPT-11 and SN-38 may also stimulate production of prostaglandins (PGs) in the rat colon (Kase et al., 1998; Yang et al., 2005), which play a key role in water and electrolyte balance, and production of pro-inflammatory cytokines, such as TNF-α, IL-1β, and IL-6 in intestinal tissue (Richardson and Dobish, 2007; Logan et al., 2008; Melo et al., 2008).

### CURRENT TREATMENTS FOR CPT-11-INDUCED DIARRHEA AND LIMITATIONS

To diagnose and determine the severity of CID, the National Cancer Institute Common Toxicity Criteria are an internationally recognized set of guidelines that assess symptoms on a scale of 0–5 (0 representing no toxicity; 5 indicating death). Standard guidelines for evaluation and management of CID were published in 2004 and updated in 2014 (Benson et al., 2004; Andreyev et al., 2014).

Generally, CPT-11-induced diarrhea can be managed by dietary modification and administration of standard antidiarrheal medications such as loperamide, the somatostatin analog octreotide, and deodorized tincture of opium. Major chemotherapeutic agents and potential underlying mechanisms for treatment of CPT-11-induced diarrhea are summarized in Table 1. However, clinical studies have demonstrated that current therapies often contribute to worsening of existing chronic gastrointestinal symptoms or induce other side effects including respiratory depression, uneven heartbeat, seizures, and neurotoxicity (Takasuna et al., 1995a; Swami et al., 2013; McQuade et al., 2016).

**Table 1:** Major chemotherapeutic agents for the treatment of CPT-11-induced diarrhea.

Chemotherapeutic agent	Mechanisms of action	Optimal dose	Side effects	Reference
Loperamide	Acts at μ-opioid receptor; slows intestinal peristalsis; anti-secretory effects through inhibition of TXA2	4 mg initial dose followed by 2 mg every 2–4 h	Constipation, flatulence, headache, nausea, dizziness, abdominal pain, worsening of already present bloating, nausea and vomiting, increasing incidents of paralytic ileus	Abigerges et al., 1994; Benson et al., 2004; Sharma et al., 2005; Richardson and Dobish, 2007; Stein et al., 2010
Octreotide	Reduces the secretion of specific gut hormones; prolongates intestinal transit time; promotes intestinal absorption of fluids and electrolytes	Persistent diarrhea (grade 1 or 2) and severe diarrhea (grade 3 or 4): 100–150 μg *via* subcutaneous injection, t.i.d. with dose escalation up to 500 μg, t.i.d.	Fast, slow, or irregular heartbeat, constipation, abdominal or stomach pain, nausea and vomiting, headache and dizziness	Gebbia et al., 1993; Barbounis et al., 2001; McQuade et al., 2016
Deodorized tincture of opium	Inhibits intestinal peristalsis; increases intestinal transit time; promotes fluid reabsorption	10–15 drops in water every 3–4 h	Constipation, nausea, vomiting, dizziness, drowsiness, itching, hives or welts, seizures, psychological and physical dependence, respiratory depression	Benson et al., 2004; Richardson and Dobish, 2007; Benyamin et al., 2008; McQuade et al., 2016


## Herbal Medicines for Treatment and Prevention of CPT-11-Induced Diarrhea

As current therapies for CPT-11-induced diarrhea are non-specific and exhibit limited efficacy, discovery of more effective modulator agents that relieve toxic side effects associated with CPT-11 treatment is essential.

Several emerging and existing therapies such as herbal formulas, plant extractions, and phytochemicals have been proven effective for the treatment and prevention of CPT-11-related diarrhea in preclinical and clinical studies. The chemical components of herbal formulas usually act simultaneously and synergistically on multiple targets in the body, representing valuable sources for the development of multi-compound and multi-target therapies to control GI toxicity (Tang and Eisenbrand, 1992; Wang et al., 2012; Swami et al., 2013).

### Huangqin Decoction

Huangqin decoction (HQD), a traditional Chinese medicine, consists of four medicinal herbs, including *Scutellaria baicalensis* Georgi, *Glycyrrhiza uralensis* Fisch*, Paeonia lactiflora* Pall, and *Ziziphus jujuba* Mill at ratio of 3:2:2:2 by dry weight (Table 2). HQD has been widely used in China for over 1800 years to treat GI syndromes that are accompanied with symptoms such as diarrhea, nausea, abdominal cramps, and vomiting (Bensky and Barolet, 1990; Wang et al., 2015). In animal experiments, co-administration with HQD (10 g/kg; b.i.d.) significantly ameliorated CPT-11-induced late-onset diarrhea in rats, but failed to prevent acute diarrhea occurring on days 1 and 2. Researchers applied a GC/MS and LC/MS metabolomics approach to detect serum metabolite changes in male Sprague-Dawley rats with or without HQD treatment and put forward that HQD mediated metabolic alterations through reversal of amino acid, lipid, and bile acid metabolic pathways (Cui et al., 2017; Wang et al., 2017). Moreover, Cui et al. (2017) demonstrated that *S. baicalensis* played a crucial role in the therapeutic effects of HQD on CPT-11-induced diarrhea.

**Table 2:** Summary of the proved effects of herbal medicines in CPT-11-induced diarrhea.

Name of formula	Botanical latin name	Ratio	Mechanisms of action	References
Huangqin decoction	*Scutellaria baicalensis* Georgi*, Glycyrrhiza uralensis* Fisch, *Paeonia lactiflora* Pall, *Ziziphus jujuba* Mill	3:2:2:2	Alters amino acids (glutamine, tryptophan, glycine, serine, and threonine), lipids, and bile acids metabolisms	Wang et al., 2015, 2017; Cui et al., 2017
PHY906	*Scutellaria baicalensis* Georgi, *Glycyrrhiza uralensis* Fisch, *Paeonia lactiflora* Pall, *Ziziphus jujuba* Mill	Unknown	Promotes regeneration of intestinal progenitor or stem cells and several Wnt signaling components; attenuates intestinal inflammation	Lam et al., 2010; Swami et al., 2013
Hange-shashin-to	*Pinellia ternate* Breit, *Scutellaria baicalensis* Georgi, *Glycyrrhiza uralensis* Fisch, *Ziziphus jujuba* Mill, *Panax ginseng* C.A. Mey, *Coptis chinensis* Franch, *Zingiber officinale* Rosc	5:2.5:2.5:2.5:2.5: 1.0:2.5	Inhibitsβ-glucuronidase; decreases colonic prostaglandin E2 production; increases water absorption	Yokoi et al., 1995; Kase et al., 1997a; Swami et al., 2013; Yamakawa et al., 2013
Sairei-to	*Radix Bupleuri Chinensis, Pinellia ternate* Breit, *Alisma orientalis* (Sam.) Juzep, *Scutellaria baicalensis* Georgi, *Panax ginseng* C. A. Mey, *Ziziphus jujuba* Mill, *Poria cocos* (Schw.) Wolf, *Polyporus umbellatus* (Pers.) Fries, *Rhizoma Areactylodis Lanceae, Cinnamomum cassia* Presl, *Glycyrrhiza uralensis* Fisch, *Zingiber officinale* Rosc	7:5:5:3:3:3:3:3: 3:2:2:1	Inhibits β-glucuronidase	Takasuna et al., 1995b; Satoh et al., 2018
Shengjiang Xiexin decoction	*Pinellia ternate* Breit, *Glycyrrhiza uralensis* Fisch, *Coptis chinensis* Franch, *Ziziphus jujuba* Mill, *Zingiber officinale* Rosc, *Scutellaria baicalensis* Georgi, *Codonopsis pilosula* (Franch.) Nannf, *Zingiberis Rhizoma Recens*	9:9:3:12:3:9:9:12	Inhibits intestinal apoptosis and β-glucuronidase; promotes intestinal cell proliferation	Deng et al., 2017; Guan et al., 2017a
Banxia Xiexin decoction	*Pinellia ternata* Breit, *Scutellaria baicalensis* Georgi, *Zingiber officinale* Rosc, *Salvia miltiorrhiza* Bunge, *Glycyrrhiza uralensis* Fisch, *Coptis chinensis* Franch, *Ziziphus jujuba* Mill	12:9:9:9:9:3:16	Unknown	Wang Y. et al., 2014, Chen et al., 2015
St. John’s wort	*Hypericum perforatum*	-	Alters CPT-11 and SN-38 pharmacokinetics; inhibits pro-inflammatory cytokines and intestinal epithelial apoptosis	Hu et al., 2005, 2006, 2007


### PHY906

PHY906, a modified four-herb pharmaceutical preparation derived from a traditional Chinese medicine formulation of HQD, was shown to be a modulator of chemotherapeutic agents in cancer therapy (Lam et al., 2010). Liu and Cheng (2012) from Professor Yung-Chi Cheng’s Team at the Yale University School of Medicine and PhytoCeutica, Inc., indicated that PHY906 was different from HQD currently available in the market owing to unique and defined procedures for preparation, standardized chemical fingerprints, and quality control (Tilton et al., 2010). Preclinical models and phase I/II clinical trials have shown that PHY906 exerted beneficial effects on outcomes of advanced liver, colorectal, and pancreatic cancers (Farrell and Kummar, 2003; Lam et al., 2010; Saif et al., 2014). Not only could PHY906 enhance therapeutic indices of a broad spectrum of anticancer drugs such as CPT-11, capecitabine, and sorafenib (Saif et al., 2014; Lam et al., 2015), but it also markedly alleviated toxicity caused by chemotherapeutic agents such as CPT-11 when co-administered with PHY906 (Saif et al., 2010; Lam et al., 2010). In a murine colon 38 allograft model, oral administration of PHY906 (50, 150, 500, or 1000 mg/kg; b.i.d.) reduced CPT-11-induced GI toxicity *in vivo* through multiple modes of action, including attenuation of intestinal inflammation through inhibition of the NF-*k*B, COX-2, and iNOS inflammatory pathways, and promotion of intestinal progenitor cell repopulation by increasing expression of several Wnt signaling components and potentiating wnt3a action (Lam et al., 2010).

A phase I, multicenter, double-blind, randomized, placebo-controlled, crossover study of PHY906 in combination with CPT-11 and 5-FU/ Leucovrin (IFL) chemotherapy involving 17 patients with advanced colorectal cancer was conducted. PHY906 decreased the overall incidence of grade 3 or 4 diarrhea in patients and contributed to lower use of the antidiarrheal drugs loperamide and lomotil. In addition, PHY906 did not alter the pharmacokinetics of CPT-11, or the CPT-11 metabolite SN-38 (Kummar et al., 2011).

### Hange-shashin-to

The Japanese Kampo medicine Hange-shashin-to is composed of seven herbs (Table 2) and has been used as an herbal formula to treat diarrhea and acute gastroenteritis (Kase et al., 1997b; Kawashima et al., 2004). In an animal experiment, Hange-shashin-to (1 g/kg; b.i.d.) and baicalin (25 mg/kg; b.i.d.), the primary flavonoid in Hange-shashin-to, exhibited protective effects against CPT-11-induced intestinal toxicity by inhibiting β-glucuronidase activity, resulting in decreased weight loss, improved anorexia, and delayed onset of diarrhea symptoms (Narita et al., 1993; Takasuna et al., 1995b).

Furthermore, repeated oral administrations of Hange-shashin-to at 125 or 500 mg/kg markedly suppressed chronic diarrheal symptoms due to CPT-11 in rats in a dose-dependent manner, which was mediated by decreasing production of colonic prostaglandin E2 (PGE2) and stimulating colonic water absorption (Kase et al., 1997a, 1998). Kase et al. (1998) revealed that three components of Hange-shashin-to, *Scutellariae radix, Glycyrrhizae radix*, and *Coptidis rhizoma* selectively inhibited cyclooxygenase-2 (COX-2) activity. Glycyrrhizin and glycyrrhetinic acid contained in *Glycyrrhizae radix*, and ginsenosides contained in *Ginseng radix* were reported to enhance colonic water absorption through promotion of corticosterone secretion.

Mori et al. (2003) from the Tochigi Cancer Center Research Group conducted a randomized comparative clinical trial involving 41 patients with non-small cell lung cancer treated with cisplatin and CPT-11. They reported that the Hange-shashin-to group (7.5 g/body; t.i.d.) showed markedly improved grades of diarrhea (*P* = 0.044) and reduced frequency of severe grade 3 or 4 diarrhea (*P* = 0.018) as compared to the control group.

### Sairei-to

Sairei-to, a traditional Japanese herbal medicine used to treat severe diarrhea and various inflammatory diseases such as rheumatoid arthritis, systemic lupus erythematodes, and nephrotic syndrome, is made from a combined formulation of twelve medicinal herbs (Ito et al., 2002; Kato et al., 2015). In a preclinical study conducted on male Wistar rats, co-administration of Sairei-to (1 g/kg; b.i.d.) alleviated CPT-11-induced delayed-onset diarrhea. The probable mechanism of action was related to the inhibition of bacterial β-glucuronidase (Takasuna et al., 1995b). Recently, Satoh et al. (2018) investigated the inhibitory effects of β-glucuronidase-treated or untreated Sairei-to on SN-38 glucuronidation in human liver microsomes. They reported that β-glucuronidase-treated Sairei-to remarkably increased baicalein, which was the key ingredient responsible for inhibition of UGT activity. Baicalein, the deglycosylation product of baicalin derived from Sairei-to, could be a pharmacokinetic regulating factor associated with SN-38-induced late-onset diarrhea *in vitro*. Similarly, Kato et al. (2015) demonstrated that Sairei-to (100, 300, or 1000 mg/kg; b.i.d.) dose-dependently attenuated 5-FU-induced diarrhea in mice during chemotherapy *via* suppression of up-regulation of inflammatory cytokines such as TNF-α, IL-1β, and TGF-β.

### Shengjiang Xiexin Decoction

Shengjiang Xiexin decoction (SXD), a traditional Chinese medicine prescribed in *Shang Han Lun*, is composed of eight Chinese herbs (Table 2) and is widely used in modern clinical practice to treat gastroenteritis, ulcerative colitis, and diarrhea (Deng et al., 2017; Guan et al., 2017b; Peng et al., 2017). In a diarrhea rat model, SXD (5, 10, or 15 g/kg/day) prevented CPT-11-induced delayed-onset diarrhea in a dose-dependent manner *via* inhibition of intestinal apoptosis and β-glucuronidase and promotion of intestinal cell proliferation (Deng et al., 2017). Using a sensitive and accurate UHPLC-MS/MS method, Guan et al. (2017a,b) demonstrated that the active constituents and metabolites derived from SXD (10 g/kg; b.i.d.) could synergistically alter the pharmacokinetics of CPT-11 to alleviate diarrhea. These alterations were associated with decreased hepatic expression of multidrug resistance-associated protein-2 (Mrp-2) and P-glycoprotein (P-gp), inhibition of CPT-11 hydrolysis to SN-38 by carboxylesterase, and improved glucuronidation of SN-38 to SN-38G. Glycyrrhizin and glycyrrhetinic acid, derived from SXD, are known inhibitors of Mrp-2 (Feng et al., 2015). The flavonoid oroxylin A, a product of metabolism of SXD by intestinal bacteria, has been reported to effectively inhibit P-gp expression (Go et al., 2009). Moreover, chrysin is likely responsible for conversion of SN-38 to SN-38G in the gastrointestinal tract through up-regulation of UGT1A1 (Guan et al., 2017b).

A randomized controlled trial was performed by Deng et al. (2017) involving 115 patients who received CPT-11 combined with 5-fluorouracil plus l-leucovorin treatment. SXD (100 mL; b.i.d.) significantly reduced delayed-onset diarrhea in UGT1A1^∗^28 or UGT1A1^∗^6 variant patients (high risk groups for CPT-11-induced hematological and GI toxicities) without affecting clinical response to chemotherapy (Deng et al., 2017).

### Banxia Xiexin Decoction

Banxia Xiexin decoction (BXD) consists of seven herbs (Table 2) and is an effective Chinese medicine prescribed for treatment of gastroenteritis, ulcerative colitis, vomiting, and diarrhea (Tian et al., 2013; Wang X. et al., 2014; Wang Y. et al., 2014). Wang Y. et al. (2014) successfully used a validated UPLC–MS/MS method to accurately quantitate 18 bioactive compounds in BXD, including flavonoids, alkaloids, and saponins. Twenty-seven Chinese patients with recurrent small cell lung cancer receiving CPT-11 chemotherapy were enrolled into a clinical study performed by Lu et al. (2018). This study confirmed that BXD was effective in preventing and controlling CPT-11-induced delayed-onset diarrhea. Delayed-onset diarrhea occurred in six patients, and 4 of 5 patients that received BXD treatment experienced relief from diarrhea symptoms. However, the overall number of patients with delayed-onset diarrhea enrolled on to this trial was relatively small. As such, the efficacy of BXD in prevention and control of delayed-onset diarrhea caused by CPT-11 should be further evaluated in a high quality RCT with a larger sample size.

### St. John’s Wort (*Hypericum perforatum*)

St. John’s wort (*Hypericum perforatum*) is one of the most commonly used herbal medicines for treatment of depression, hypertension, and inflammation (Di Carlo et al., 2001; Fiebich et al., 2001; Rahimi and Abdollahi, 2012). Over two dozen compounds were identified in St. John’s wort, among which hyperforin, hypericin, and quercetin were the major active components (Hu et al., 2007). A preliminary study in a rat model clearly demonstrated that oral pretreatment with St. John’s wort (400 mg/kg/day) for 8 consecutive days significantly reduced both early- and late-onset diarrhea induced by use of CPT-11 (Hu et al., 2005, 2006). This protective effect was partially attributed to modulation of CPT-11 and SN-38 pharmacokinetics by the main constituents of St. John’s wort (Hu et al., 2005, 2007). Additionally, co-administration of St. John’s wort and CPT-11 prevented intestinal histological damage and diarrhea *via* inhibition of intestinal epithelial apoptosis and pro-inflammatory cytokine expression in the intestine, including IFN-γ, IL-6, IL-1β, and TNF-α (Hu et al., 2006). In an unblinded, randomized crossover study on 5 cancer patients, oral treatment with 900 mg/day St. John’s wort for 18 days attenuated CPT-11-induced diarrhea by decreasing plasma levels of SN-38 (Mathijssen et al., 2002).

### Others

The flavonoid-rich fraction (100 mg/kg/day; p.o.) from *Bauhinia forficata* leaves, collected at Itajaí, Santa Catarina (Brazil), has been shown to partially mitigate intestinal mucositis in mice, which is characterized by diarrhea and histological damage without interfering with anticancer efficacy of CPT-11. Probable mechanisms were the maintenance of protective factors in the duodenum, the augmented viability of GSH, and the increased release of pro-inflammatory cytokines, particularly TNF-*α* (Cechinel-Zanchett et al., 2018). de Alencar et al. (2017) reported that latex proteins (1, 5, or 50 mg/kg/day; i.v.) from *Calotropis procera* prevented intestinal damage and persistent diarrhea in a dose-dependent manner in CPT-11-treated animals by down-regulating key pro-inflammatory mediators such as COX-2, TNF-*α*, IL-1β, iNOS, and NF-*κ*B.

Hesperetin, a widely distributed flavonoid in many plant fruits and flowers, and foods of plant origin, has been reported to have powerful antioxidant, cholesterol-lowering, and anti-inflammatory pharmacological properties (Parhiz et al., 2015; Polat et al., 2018). Oral administration of hesperetin (20 or 100 mg/kg/day) exerted a remarkable anti-diarrhea effect, blocking 80% of severe and 100% of mild diarrhea cases observed in mice given CPT-11. The mechanism of action of hesperetin was attenuation of intestinal damage caused by SN-38 accumulation *via* selective inhibition of intestinal carboxylesterase (Yu et al., 2018).

## Conclusion and Future Prospects

CPT-11 is a potent anticancer agent for the treatment of colorectal, non-small cell lung cancer, and various other cancers and has been used for almost two decades. However, severe and persistent diarrhea greatly compromise the efficacy and safety of CPT-11-based chemotherapy. Although effective therapies for acute diarrhea resulting from enhanced intestinal motility caused by the inhibition of cholinesterase are available, there are no effective treatments and/or prevention strategies for CPT-11-induced delayed-onset diarrhea.

As shown in this review, preclinical and clinical studies have indicated that several herbal formulations, medicine preparations, plant extracts, and phytoconstituents have potential to prevent or attenuate chronic diarrheal symptoms during CPT-11-based chemotherapy. The proposed mechanisms of action of herbal medicines for treatment of delayed-onset diarrhea are diverse, mainly acting on metabolism of CPT-11, which occurs through a number of enzymes, metabolites, and transporters.

Herbal prescriptions are often composed of more than one herb, and chemical components within a single herb can be complex and varied. Further investigations should focus on characterization of bioactive compounds responsible for the protective effects against CPT-11-induced diarrhea and in-depth studies of mechanisms of action. Although some progress has been made in basic research and clinical application of herbal medicines for treatment of CPT-11-related diarrhea, many clinical trials were not well-designed with small sample sizes, poor methodological control, and short durations of therapy and follow-up. Larger methodologically sound, randomized controlled trials should be performed in the future.

## Author Contributions

All listed authors fulfill the requirements for authorship and have approved the final version of the manuscript. LT and HD prepared the manuscript. HD conceived the idea, reviewed the drafts, and provided important information for the completion of this manuscript. XL, LW, YX, and XZ contributed to the revision of the manuscript.

## Conflict of Interest Statement

The authors declare that the research was conducted in the absence of any commercial or financial relationships that could be construed as a potential conflict of interest.
